# Differential expression of a novel serine protease homologue in squamous cell carcinoma of the head and neck

**DOI:** 10.1054/bjoc.2000.1586

**Published:** 2001-01

**Authors:** J C Lang, D E Schuller

**Affiliations:** Department of Otolaryngology and Comprehensive Cancer Center, 1248 James Cancer Hospital and Solove Research Institute, The Ohio State University, 300 West 10th Avenue, Columbus, Ohio, 43210, USA

**Keywords:** serine protease, *DESC1*, squamous cell carcinoma, head and neck, differential expression, chromosomal location

## Abstract

Differential gene expression between squamous cell carcinoma of the head and neck and matched normal tissue was studied by utilizing Representational Difference Analysis. Using this methodology, a novel gene, *DESC1* was isolated. *DESC1* possesses strong identity to the serine protease super-family. Comparison of *DESC1* expression between primary squamous cell carcinoma and matched normal tissue shows that the level of *DESC1* expression is reduced or absent in 11/12 SCC tissue specimens when compared to specimens of matched normal tissue. Tissue-specific expression studies further show that *DESC1* expression can only be detected in tissues derived from the head and neck, and in skin, prostate and testes. Cell line studies demonstrate that *DESC1* expression is epithelial-specific. Chromosomal localization studies indicate that *DESC1* is located on the long arm of chromosome 4 at position q12–13. © 2001 Cancer Research Campaign http://www.bjcancer.com


*British Journal of Cancer* (2001) **84**(2), 237–243
© 2001 Cancer Research Campaign
doi: 10.1054/ bjoc.2000.1586, available online at http://www.idealibrary.com on http://www.bjcancer.com

### Differential expression of a novel serine protease homologue in squamous cell carcinoma of the head and neck

**JC Lang and DE Schuller**

The Ohio State University, Department of Otolaryngology and Comprehensive Cancer Center, 1248 James Cancer Hospital and Solove Research Institute, 300 West 10th Avenue, Columbus, Ohio 43210, USA

**Summary** Differential gene expression between squamous cell carcinoma of the head and neck and matched normal tissue was studied by utilizing Representational Difference Analysis. Using this methodology, a novel gene, *DESC1* was isolated. *DESC1* possesses strong identity to the serine protease super-family. Comparison of *DESC1* expression between primary squamous cell carcinoma and matched normal tissue shows that the level of *DESC1* expression is reduced or absent in 11/12 SCC tissue specimens when compared to specimens of matched normal tissue. Tissue-specific expression studies further show that *DESC1* expression can only be detected in tissues derived from the head and neck, and in skin, prostate and testes. Cell line studies demonstrate that *DESC1* expression is epithelial-specific. Chromosomal localization studies indicate that *DESC1* is located on the long arm of chromosome 4 at position q12–13. © 2001 Cancer Research Campaign http://www.bjcancer.com

**Keywords**: serine protease; *DESC1*; squamous cell carcinoma; head and neck; differential expression; chromosomal location

Investigation of the genetic alterations underlying squamous cell carcinoma of the head and neck (SCCHN) has recently allowed identification of a number of key genes frequently altered during disease progression. Many of these studies have concentrated on the role of oncogenes and tumour suppressor genes in the disease. Thus, oncogenes c-myc (Field et al, 1989), EGFR (Ke et al, 1998), HER-2/neu (Xia et al, 1997) and cyclin D1 (Michalides et al, 1995), and tumour suppressor genes *p53* (Somers et al, 1992) and *p16* (Reed et al, 1996; Lang et al, 1998), demonstrate disease-specific alterations during genesis of SCCHN. However, a number of studies have implicated additional chromosomal loci in the disease, suggesting the existence of additional unidentified genes, which may be targets for inactivation during SCCHN development (Nawroz et al, 1994; Califano et al, 1996; Loughran et al, 1997; Bockmuhl et al, 1998).

In this study, we have performed Representational Difference Analysis (RDA) (Diatchenko et al, 1996) in order to identify novel genes expressed in normal epithelium but absent from SCC. This study has allowed isolation of a differentially expressed novel gene named DESC1. DESC1 possesses strong identity to members of the serine protease superfamily, and shows highest degree of homology with Human Airway Trypsin-like protease (HAT; Yamaoka et al, 1998).

### MATERIALS AND METHODS

### RNA isolation

Normal human keratinocytes (NHEK-Neo, NHEK-Ad) and prostate epithelial cells (PrEC) were cultured in keratinocyte growth medium (KGM) or prostate epithelial cell growth medium (PrEGM) (Clonetics, San Diego, CA) respectively and exponentially growing cells harvested. Tissue samples were provided by the Cooperative Human Tissue Network, funded by the National Cancer Institute. Total RNA was extracted from cell lines and tissue as previously described (Gramza et al, 1995). Normal prostate and testes RNA was obtained from CLONTECH (Palo Alto, CA) and normal skin RNA from Invitrogen (Carlsbad, CA). Polyadenylated RNA was selected utilizing an Oligotex kit (Qiagen, Santa Clarita, CA).

### Representational difference analysis and gene cloning

Representational Difference Analysis (RDA) was performed by utilizing PCR-Select cDNA subtraction methodologies (CLONTECH, Palo Alto, CA) precisely as described in the manufacturer's protocol. PCR amplification products obtained were cloned directly into mammalian expression vector pCMV-Script (Stratagene, La Jolla, CA). Additional DESC1 5′ and 3′ sequence not present in CMV-Script clone, was obtained by 5′ and 3′ RACE analysis utilizing both 5′ and 3′ RACE systems (Gibco BRL, Gaithersburg, MD). A full-length *DESC1* cDNA clone (pDESC1) was constructed following contig analysis of sequence obtained from pCMV-Script, 5′ and 3′ RACE clones. pDESC1 was generated by cloning of the DESC1 RT-PCR amplification product derived from normal human skin RNA (Invitrogen, Carlsbad, CA) into TOPO TA cloning vector pcDNA3.1/V5/His-TOPO (Invitrogen, Carlsbad, CA) utilizing DESC1 primers D11 and D12, which delimit the known DESC1 sequence.

### Inducible expression and Western analysis of DESC1

The entire *DESC1* cDNA was also cloned into inducible expression vector pIND TOPO TA. This vector was transiently transfected into

*Received 15 February 2000*
*Revised 18 September 2000*
*Accepted 18 October 2000*

*Correspondence to:* JC Lang modified 293 cells (EcR-293) containing a heterodimeric ecdysone and retinoid X receptor. Induction of *DESC1*, linked to a paramyxovirus V5 epitope tag, was accomplished by addition of Ponasterone A to the cell medium as described in the manufacturer's protocol (Invitrogen, Carlsbad, CA). 72 hours post transfection, cell lysates were prepared, 20 µl lysate run on a 10% NuPAGE gel, and blotted using an XCell II Blot Module (Invitrogen, Carlsbad, CA). Presence of the recombinant *DESC1* polypeptide was confirmed by WesternBreeze (Invitrogen, Carlsbad, CA) chemiluminescence detection, utilizing an antibody to the V5 epitope.

### RT-PCR amplification

1.0 µg of total RNA was used for first strand cDNA synthesis in a total volume of 25 µl and reactions otherwise performed according to manufacturer's instructions (ProSTAR, Stratagene, La Jolla, CA). PCR amplification was performed in the presence of 2 units Taq 2000 DNA polymerase (Stratagene, La Jolla, CA), with reaction conditions: 10 mM Tris-HCl (pH 8.8), 50 mM KCl, 1.5 mM $MgCl_2$, 0.001% gelatin, 1µM each primer, 200 µM dNTPs, and where appropriate, 0.25 µl [$\alpha^{32}$P]dCTP (3000 Ci mmol$^{-1}$) in a final volume of 25 µl. Separate reactions were performed for each primer pair with reaction conditions; 96˚C 3 min followed by 94˚C 30 s, 55˚C 30 s, 72˚C 1 min for 31 cycles (HPRT) or 33 cycles (*DESC1*) and a final 5 min extension at 72˚C. PCR amplification of full-length *DESC1* was performed utilizing the above cycling conditions, with an additional 1 min extension time for each cycle, and using the Advantage HF PCR kit (CLONTECH Laboratories Inc, Palo Alto, CA). PCR samples were then run through 2% agarose gels and presence of amplified product and correct product size verified by ethidium bromide fluorescence in the presence of 100 bp size markers (Gibco BRL, Gaithersburg, MD). Where appropriate, PCR products generated were electroblotted using a Bio-Rad Semi-Dri Electroblotter SD and transferred at 12 V/110 mA for 10 min. The membrane was removed, UV crosslinked and exposed to BioMax film (Eastman Kodak, Rochester, NY). Primers utilized in PCR reactions comprise: hypoxanthine phosphoribosyl transferase (HPRT) primers HPRT1, 5′-GTAATGACCAGTCAACAGGGGAC-3′ and HPRT2, 5′-CCAGCAAGCTTGCGACCTTGACCA-3′ and *DESC1* primers D3, 5′-TCACTGTTCATTATGTGAGATATAATCA-3′; D4, 5′-CACCATTGATTCAAGTCTCTGGCTCAT-3′; D10, 5′-CCTGTTCCCTACACAAATGCAGTAC-3′; D11, 5′-TGCATC AAGCAAACAGTTTATTGAGATC-3′; D12, 5′-TGACTTGGATGTAGACCTCGACCTTCAC-3′ and D18, 5′-GGAATAGTGAGCTCGGGAGATG-3′. Location of primers on the *DESC1* sequence is shown in Figure 1A. PCR amplification of the HPRT gene was performed as a control to demonstrate equal loading and to determine integrity of RNA. Primers were designed by computer analysis (Oligo 4.0; NBI, Hamel, MN) of available DNA sequence for each gene and, with the exception of primer set D11, D18, are intron-spanning precluding PCR amplification of any residual DNA present in RNA samples. Optimum cycle number for PCR amplification was pre-determined for each primer set using a mixture of RT reactions from 10 random normal and tumour samples (data not shown). This step is necessary to ensure that PCR amplification remains in the linear range and that production of PCR product does not plateau. Under reaction conditions used, quantity of PCR product is therefore directly proportional to the amount of radioactivity incorporated into the DNA.

### Northern hybridization

Northern blot containing SCC RNA was generated by electrophoresis of 25 µg total RNA on a 1.5% glyoxal agarose gel according to manufacturer's instructions (NorthernMax-Gly, Ambion, Austin Tx). Human Cancer Cell Line Multiple Tissue Northern Blot, Human Multiple Tissue Northern Blot and Human Multiple Tissue Northern Blot II were purchased from CLONTECH Laboratories Inc (Palo Alto, CA). Human Normal Tissue Blot III was obtained from Invitrogen (Carlsbad, CA). Blots were hybridized with [$\alpha^{32}$P]dCTP-labelled DESC1 cDNA probe spanning 581 nucleotides of the DESC1 coding sequence (nucleotides 165–746) (Figure 1A), or control β-actin cDNA (CLONTECH Laboratories Inc, Palo Alto, CA) using ExpressHyb solution (CLONTECH Laboratories Inc, Palo Alto, CA), according to manufacturer's protocol. Blots were then washed in 0.5 × SSC, 0.1% SDS for 30 min at RT, followed by 0.1 × SSC, 0.1% SDS for 1 h at 50˚C with 2 changes of solution and exposed to BioMax film.

### DNA sequencing

DNA sequence of *DESC1* was determined by cycle sequencing using a Thermo Sequenase system (Amersham, Cleveland, OH), followed by electrophoresis using the CastAway sequencing system (Stratagene, La Jolla, CA) according to manufacturer's instructions.

### Chromosomal localization of *DESC1*

Chromosomal mapping of *DESC1* was performed by use of a Somatic Cell Hybrid Panel (Oncor, Gaithersburg, MD), with hybridization conditions for *DESC1* probe as described above. Chromosomal mapping of *DESC1* was additionally performed using the Genebridge 4 Radiation Hybrid Panel (Research Genetics Inc, Huntsville, AL) by PCR amplification using primers D11 and D18. The chromosomal location of *DESC1* was then determined by accessing the Whitheead Institute/MIT Center for Genome Research radiation hybrid map of the human genome.

### RESULTS

### Isolation of *DESC1* by representational difference analysis

Specimens for analysis comprising primary carcinoma, metastatic node and matched normal tissue were obtained from an individual who presented with a primary SCC of the tongue which was metastatic to regional neck nodes. Representational Difference Analysis (RDA) was performed on mRNA isolated from the normal oral tissue (buccal mucosa) and from an SCC-positive metastatic neck node. SCC-positive nodal tissue was chosen in preference to primary carcinoma, precluding the possibility of contamination of the carcinoma sample with normal oral epithelium. RDA was performed with carcinoma RNA as driver in the reaction, allowing selection of genes expressed in normal tissue but not in tumour tissue. 0.5% of the RDA final reaction was subject to PCR amplification and PCR products cloned into mammalian expression vector pCMV-Script.

© 2001 Cancer Research Campaign

### Sequence analysis and characterization of *DESC1*

All recombinant clones were screened for inserts by PCR analysis and positive clones subject to sequence analysis using vector-specific T3 primers. BLASTN sequence analysis was then performed, using the public domain GenBank sequence database. One recombinant clone C35, carrying a 581 bp insert, was shown to represent a novel sequence, and possesses an open reading frame spanning the full length of the clone. The putative gene represented by this clone was designated *D*ifferentially *E*xpressed in *S*quamous Cell *C*arcinoma Gene 1 (*DESC1*). C35 was devoid of consensus polyadenylation signals. In order to obtain the 3′ end of the gene, 3′ RACE analysis was performed on the remaining normal tissue mRNA used previously for RDA analysis. Sequence analysis of 3′ RACE products allowed identification of two consensus polyadenylation signal sequences separated by 633 bp (Fig. 1A) and located at nucleotide positions 802 – 808 and 1441–1446. In order to obtain additional 5′ sequence, 5′ RACE was also performed. A full-length cDNA clone (*pDESC1*) was then generated by cloning of the *DESC1* RT-PCR amplification product derived from normal human skin RNA. The resulting full-length *DESC1* cDNA sequence is 1471 nucleotides in length (Genbank accession #AF064819) and is represented in Figure 1A. The DESC1 sequence was used to query the GenBank database. This analysis demonstrates strong sequence homology between *DESC1* and a recently described serine protease, Human Airway Trypsin-like Protease (HAT) (Figure 1B). Sequence identity between the two genes at the amino acid level is 38% overall, and 51% when the serine protease catalytic domain only is compared (*DESC1* residues 191–422). This information suggests that *DESC1* is a novel member of the serine-protease gene family.

*DESC1* contains a predicted open reading frame (ORF) comprising a minimum of 422 amino acids (Figure 1B) with putative initiation codon at nucleotide position 56 (based on sequence comparison with HAT) and termination codon at nucleotide position 1322. However, it is possible that the ORF may potentially extend 5′ of nucleotide 56, since nucleotides 53–55 also encode a methionine residue and since no in-frame termination codons have been identified upstream of the putative initiation codon in the gene sequence so-far obtained. That *DESC1* is indeed capable of encoding a polypeptide is demonstrated in Figure 2. The *DESC1* gene was linked to an inducible expression vector (pIND TOPO TA), and expression induced in a modified 293 cell line (EcR-293) containing a heterodimeric ecdysone and retinoid X receptor. In this system, induction of *DESC1* was accomplished via the addition of a synthetic analogue of ecdysone (Ponasterone A) to EcR-293 cells transiently transfected with recombinant vector *DESC1*/pIND TOPO TA. Binding of the heterodimeric receptor, in the presence of Ponasterone A, to a hybrid response element controlling transcription of *DESC1* in the recombinant vector, subsequently induces *DESC1* expression. The DESC1 polypeptide is produced as a fusion protein with a carboxy-terminal tagged epitope (paramyxovirus V5). Western blotting using an anti-V5 antibody confirms the presence of a 52 kDa polypeptide comprising DESC1 (47 kDa) and the V5 epitope tag (5 kDa).

The *DESC1* polypeptide possesses four regions of conserved homology with members of the serine protease gene family which further suggest that *DESC1* is a serine protease. These regions comprise the 3 domains surrounding the conserved amino acids of the catalytic triad known to be essential for function of the serine proteases (Cheah et al, 1990), histidine (residue 231), aspartic acid (residue 276) and serine (residue 372). The fourth domain comprises the catalytic cleavage site, cleaving between residues 190 (arginine) and 191 (isoleucine) (Figure 1B). In addition, *DESC1* contains a hydrophobic region spanning residues 18–37 which represents a putative transmembrane domain. Thus *DESC1* may, similar to HAT, be a transmembrane-spanning serine protease possessing an extracellular COOH-terminal catalytic region.

### Expression studies of *DESC1*

Figure 3 demonstrates that *DESC1* is expressed at a high level in the original normal tissue sample used for RDA analysis, but, in contrast, is expressed at a very low level in the primary tongue carcinoma and is absent from the metastatic nodal tissue derived from the same individual. Residual expression of *DESC1* in the primary carcinoma may however result from low-level contamination of the tumour tissue with a small amount of surrounding normal cells.

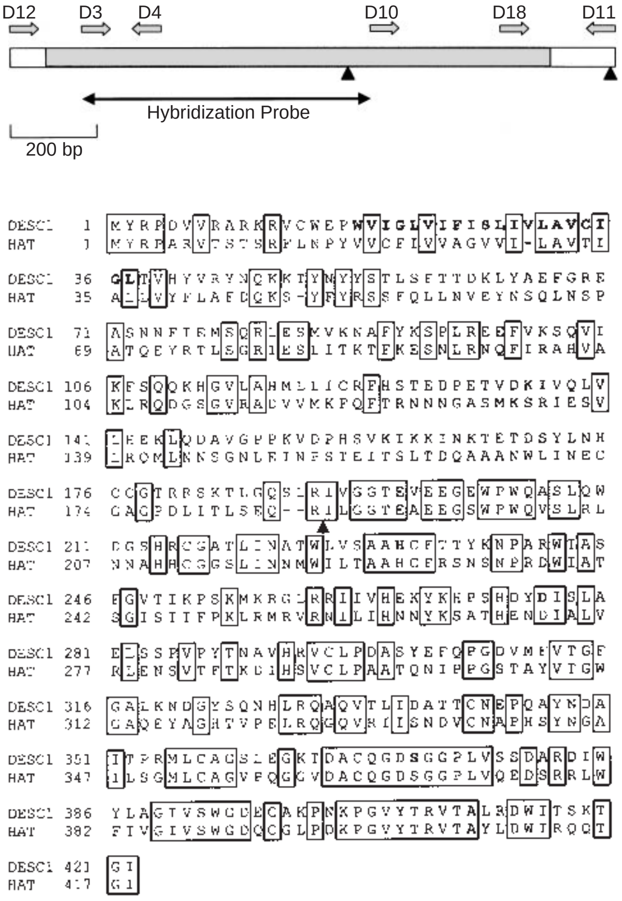

**Figure 1** (**A**) Schematic representation of *DESC1*. Putative open reading frame is shown as shaded box. Position of primers used for PCR amplification is shown as shaded arrow. Location of hybridization probe is shown as solid arrow. Location of polyadenylation signal is shown as solid triangle. (**B**) Derived amino acid sequence for *DESC1* and comparison with serine protease HAT. Identical amino acids are boxed. Residues predicted to represent conserved amino acids of the catalytic triad (His 231, Asp 276 and Ser 372) are shown in bold, as are residues 18 (Trp) through 37 (Leu) representing a putative transmembrane-spanning hydrophobic domain. Suggested location of catalytic cleavage site is shown as vertical arrow

*© 2001 Cancer Research Campaign* 

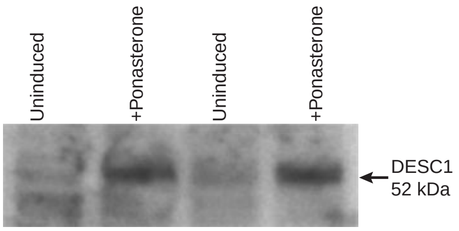

**Figure 2** Inducible expression of *DESC1* polypeptide. Western blot showing expression in 293 cells of 52 kd polypeptide consisting of *DESC1* (47 kDa) and tagged V5 epitope (5 kDa) after induction with Ponasterone. Lysates from duplicate flasks are shown

Since *DESC1* was isolated from normal buccal mucosa tissue and not from normal tongue (site of primary carcinoma), it was especially important to further determine whether *DESC1* is differentially expressed consistently between normal tissue and carcinoma in patient specimens, other than the parental tissue used for RDA analysis. Figure 4 shows RT-PCR analysis of expression of *DESC1* in 10 SCC specimens, and matched normal tissue, selected from diverse sites in the head and neck region. The results show lower levels of expression of *DESC1* in 9/10 primary carcinomas relative to the level of expression in matched normal tissue. All normal tissue specimens were harvested from clinically appearing normal tissue located at least 3 cm from the tumour margin. Pathological evaluation of the normal tissue was performed. The relative levels of expression of *DESC1* in normal tissue samples also varies between specimens, while the expression level of control gene *HPRT* remains constant.

Since *DESC1* was isolated from normal oral mucosal tissue, which additionally contains cells of non-epithelial origin, it was essential to determine whether *DESC1* expression was epithelial-specific. The expression level of *DESC1* was therefore determined in epithelial cell lines and tissue. Figure 5 demonstrates that *DESC1* is expressed in normal neonatal (NHEKNeo) and adult (NHEKAd) keratinocytes, and in normal prostate (PrEC) epithelial cells, confirming epithelial-specific expression of the gene. Figure 5 also demonstrates *DESC1* expression in human skin and additionally in prostate tissue, supporting the detection of *DESC1* in the epithelial cell lines. Expression of *DESC1* is also seen in testes tissue but at a lower level. In contrast, no expression of *DESC1* was detectable in COLO320 (colon carcinoma), MCF7 (breast carcinoma), A431 (epidermoid carcinoma), nor in cell lines of haematopoietic origin, K562 and U937. To date, expression of *DESC1* has not been detected in any cell line of non-epithelial origin.

To further characterize the tissue-specific expression pattern of *DESC1*, Northern analysis was performed on a squamous cell carcinoma specimen, matched normal oral tissue, and other diverse human tissue samples and cell lines. The results are shown in Figure 6 and demonstrate a high degree of tissue specificity of *DESC1* expression. Northern analysis, utilizing total RNA, shows expression of *DESC1* in normal oral, gingival tissue but not in gingival squamous cell carcinoma from the same patient (Figure 6A). Figure 6A also shows faint, but detectable *DESC1* expression in squamous cell carcinoma cell line SCC4, derived from a tongue carcinoma, but no expression in SCC25. *DESC1* is also detectable in normal tonsil tissue (Figure 6B) using a total RNA blot. Under these conditions no significant level of *DESC1* expression can be detected in any other tissue examined including prostate and testes. Figure 6C demonstrates, however, that when Northern analysis is performed with a higher degree of sensitivity, utilizing polyadenylated RNA rather than total RNA, *DESC1* expression can again clearly be detected in prostate and testes. All other human tissues shown, and additionally heart, brain, placenta, lung, liver, skeletal muscle and kidney (Human Multiple Tissue Northern Blot, CLONTECH Laboratories Inc, Palo Alto, CA) (not shown) do not demonstrate detectable levels of expression of *DESC1* suggesting that this gene is strongly tissue-specific in its pattern of expression. In addition, expression of *DESC1* can be detected at a very low level in pancreas (data not shown). A panel of human cancer cell lines (Human Cancer Cell Line Multiple

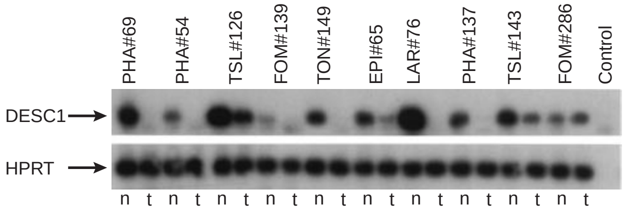

**Figure 4** RT-PCR analysis of *DESC1* expression in SCCHN and matched normal tissues. Primers utilized: D3, D4. Size of RT-PCR product 149 bp. Low molecular weight band seen in reverse transcriptase-negative control lane and some sample lanes represents unincorporated [$\alpha^{32}$P]dCTP. PHA, pharynx; TSL, tonsil; FOM, floor of mouth; TON, tongue; EPI, epiglottis; LAR, larynx

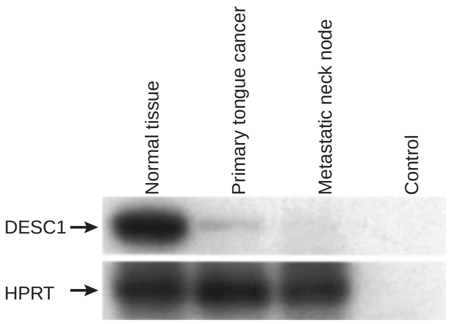

**Figure 3** *DESC1* expression in normal tissue and metastatic neck node used for RDA analysis and in primary carcinoma from the same patient. Primers utilized: D10, D11. Size of RT-PCR product, 555 bp

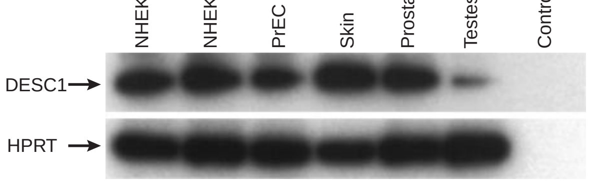

**Figure 5** Expression of DESC1 in epithelial cell lines and human tissues. RT-PCR analysis using total RNA derived from human epithelial cell lines and tissues. Primers utilized, D10 and D11

*© 2001 Cancer Research Campaign*

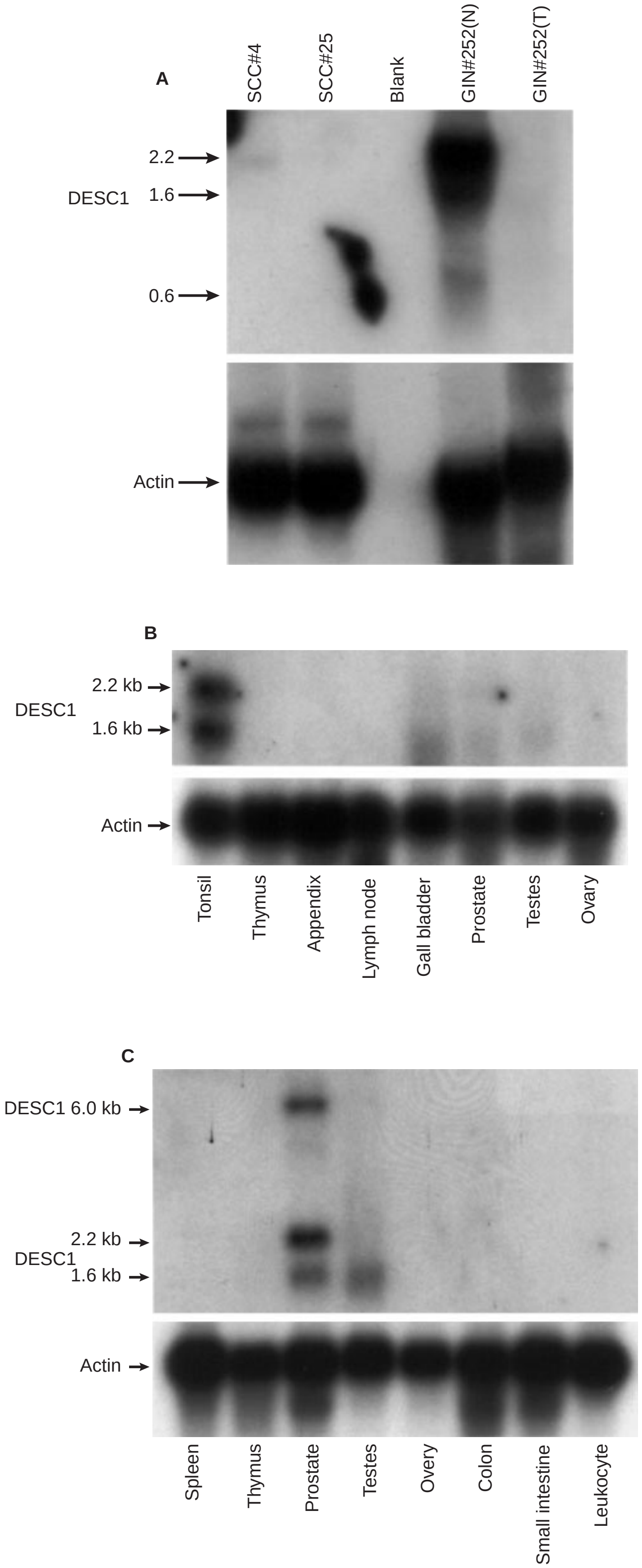

**Figure 6** Expression of *DESC1* in multiple human tissues and cell lines. Northern analysis using total RNA isolated from: (**A**) SCCHN-derived cell lines (SCC4 and SCC25), a gingival carcinoma (GIN#252(T)) and matched normal tissue (GIN#252(N)). Molecular weight markers used: Millennium Markers (Ambion, Austin, TX). (**B**) Normal human tissues (Human Normal Tissue Blot III) and (**C**) Polyadenylated RNA isolated from normal human tissues (Human Multiple Tissue Northern Blot II)

Tissue Northern Blot, CLONTECH Laboratories Inc, Palo Alto, CA), including haematopoietic cells HL-60, K-562, MOLT-4 and RAJI, colon carcinoma SW480, lung carcinoma A549, melanoma G361 and HeLa cells also lack detectable expression of *DESC1* (data not shown). The results show one predominant transcript of 2.2 kb in normal matched tissue from SCC specimen #252 (Figure 6A), and two other transcripts of 1.6 and 0.6 kb in size, expressed at a lower level. The 6.0 kb transcript found in prostate mRNA was not detectable in normal oral tissue specimen #252 (Figure 6A) nor in RNA isolated from normal tonsil tissue (Figure 6B). However, the analyses were performed on total RNA only. Whether the 6.0 kb transcript is co-expressed with the other *DESC1*-specific transcripts in oral epithelium, or is specific to prostate tissue, awaits Northern analysis of purified, polyadenylated RNA isolated from oral epithelial tissue. *DESC1* expression in normal tonsil demonstrates only the 2.2 and 1.6 kb transcripts, which, in contrast, are expressed at equal intensities. *DESC1* expression in the prostate shows expression of the 2.2 and 1.6 kb transcripts and an additional transcript of 6.0 kb, but again no detectable expression of the 0.6 kb transcript. The 1.6 kb transcript alone is detectable in testes and this may account for the lower level of *DESC1* expression detected in the testes by RT-PCR analysis (Figure 5).

### *DESC1* is located on chromosome 4

*DESC1* was hybridized to a human/rodent somatic cell hybrid mapping panel. *DESC1* hybridizes only to the lane containing human chromosome 4 and localizes *DESC1* to this chromosome (Figure 7). Further analysis utilizing the Genebridge 4 Radiation Hybrid Mapping Panel localizes *DESC1* to the long arm of chromosome 4, positioned 20.21 cR from marker WI-5548, and between markers D4S1619 and WI-7844. D4S1619 and WI-7844 have been mapped by FISH analysis to 4q12 and 4q13 respectively and *DESC1* can therefore be placed at 4q12–13 within a region approximately 10Mb in size (personal communication, Dr Mariano Rocchi, Istituto di Genetica, Italy).

### DISCUSSION

In this report we describe characterization of *DESC1*, a novel human gene encoding a serine protease homologue. *DESC1* expression has been detected in all normal specimens of head and neck-derived epithelium so far examined. In contrast, expression is reduced or absent in matched specimens of squamous cell carcinoma obtained from the same group of patients. The expression level of *DESC1* in normal tissue samples varies between specimens, even when the tissue samples are taken from the same anatomical site, for example, pharyngeal specimens #54, #69, and

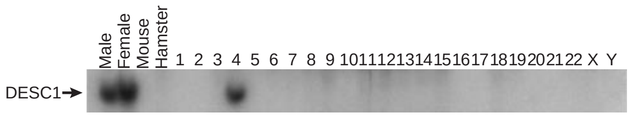

**Figure 7** Chromosomal localization of *DESCI.* Southern blot demonstrating hybridization of *DESCI* cDNA (nucleotides 165–746) to a Somatic Cell Hybrid Panel. Human chromosome (1–22, X and Y) present in each hybrid is shown. Lanes 1 and 2 show total human DNA as positive control. Lanes 3 and 4 comprise total DNA from parental rodent cells used to produce hybrids

*© 2001 Cancer Research Campaign*

#137. To further investigate this observation, the level of expression of *DESC1* in normal oral tissue samples from healthy individuals with no evidence of cancer should be determined. Such analysis will establish whether the observed variation in expression of *DESC1* in normal epithelial tissue from cancer patients is representative of premalignant or hyperproliferative changes in clinically appearing normal epithelium, or simply the result of normal variation. The lower, but observable expression of *DESC1* shown in some primary SCC specimens may represent lower overall expression in carcinoma tissue or, alternatively, may represent lack of tumour-specific expression and a degree of contamination of tumour specimens with normal tissue. In support of the former, a low level of expression of *DESC1* can be detected in cell line SCC4, derived from a tongue carcinoma (Figure 4A). Thus, it is possible that *DESC1* expression may be present in a reduced amount in many of the SCCHN tumours. It is also possible that variations in *DESC1* expression may, in part, result from differences in the degree of tumour differentiation. Our preliminary observations suggest that expression of *DESC1* does increase during enforced differentiation of normal epithelial cells (data not shown).

Northern analysis has allowed identification of 4 *DESC1* transcripts, 6.0, 2.2, 1.6 and 0.6 kb in length. The additional identification of an internal consensus polyadenylation signal (nucleotides 802–807) suggests that the transcriptional organization of this locus is complex. The RT-PCR analysis of *DESC1* expression shown in Figure 5 utilizes primers D10 and D11, specific to the 3′ end of *DESC1*. This region of the gene is downstream from, and therefore does not encompass, the internal consensus polyadenylation site, although it does contain part of the conserved catalytic domain of the gene (Figures 1A and 1B). Since this analysis allows detection of *DESC1* expression in testes, and since, by Northern analysis, testes exhibit only the smaller 1.6 kb transcript, the data is consistent with identification of the 1.6 kb RNA as the transcript which encodes the *DESC1* polypeptide comprising the serine protease. A second 3′ RACE product isolated demonstrates processing of a *DESC1* RNA at the internal polyadelylation site (data not shown). This transcript terminates at nucleotide 823 and demonstrates that the internal polyadenylation site is functional. This alternative *DESC1* transcript has not yet been further investigated, and it is unknown which transcript it may represent. The transcript cannot encode the putative *DESC1* serine protease and is unlikely to encode an alternative reading frame, due to the presence of multiple upstream termination codons in the sequence so far obtained. However, should this alternative transcript comprise the 2.2 or 6.0 kb RNA, the remaining 5′ sequence of this RNA, yet to be determined, may be sufficient in length to encode another polypeptide. Expression of the 1.6 and 2.2 kb *DESC1* transcripts appear equi-molar in normal tonsil tissue (Figure 6B), yet, the 2.2 kb transcript predominates in the matched normal tissue sample from SCC#252 (Figure 6A). The reason for this difference is not evident, but further suggests that detailed mapping of the alternative *DESC1* transcripts should be pursued.

The role of proteases, including serine proteases, in cancer development is well documented, and demonstrates an association between cancer development and activation of proteases (Liotta and Stetler-Stevenson, 1991). Less well described, is the inactivation of proteases associated with cancer development. However, it is interesting that a second, epithelial-specific serine protease, *NES1*, has been shown to be down-regulated during breast cancer development and it has been suggested that this gene may possess the functional properties of a tumour suppressor gene (Goyal et al, 1998). The results of our study suggest that *DESC1* may similarly be down-regulated during the development of SCCHN, although we have no direct evidence to suggest that *DESC1* also possesses the ability to function as a tumour suppressor. *DESC1* is highly expressed in primary, normal human keratinocytes, epithelial cells previously shown to be capable of malignant conversion (Rhim et al, 1985). Functional studies are therefore underway in our laboratory to determine whether expression of *DESC1* is altered during immortalization and transformation of normal human keratinocytes and whether enforced expression of *DESC1* affects the growth characteristics of cells already transformed. Although the precise role of *DESC1* in the development of SCCHN is unknown, the observation of altered *DESC1* expression in the majority of tumours suggests that this gene may represent a useful marker for squamous cell carcinoma of the head and neck.

### ACKNOWLEDGEMENTS

This work was supported by grants from the National Institutes of Health, CA63134 (National Cancer Institute) (JCL) and P01 DE12704 (National Institute for Dental and Craniofacial Research) (JCL and DES) and by grant 2P30CA16058 from the National Cancer Institute.

### REFERENCES

Bockmuhl U, Wolf G, Schmidt S, Schwendel A, Jahnke V, Dietel M and Peterson I (1998) Genomic alterations associated with malignancy in head and neck cancer. *Head Neck* **20**: 145–151

Califano J, Van Der Riet P, Westra W, Nawroz H, Clayman G, Piantadosi S, Corio R, Lee D, Greenberg B, Koch W and Sidransky D (1996) Genetic progression model for head and neck cancer: implications for field cancerization. *Cancer Res* **56**: 2488–2492

Cheah K-C, Leong L E-C and Porter AG (1990) Site directed mutagenesis suggests close functional relationship between a human rhinovirus 3C cysteine protease and cellular trypsin-like serine proteases. *J Biol Chem* **265**: 7180–7187

Diatchenko L, Lau YFC, Campbell AP, Chenchik A, Moqadam F, Huang B, Lukyanov S, Lukyanov K, Gurskaya N, Sverdlov ED and Siebert PD (1996) Suppression subtractive hybridization: A method for generating differentially regulated or tissue-specific cDNA probes and libraries. *Proc Natl Acad Sci USA* **93**: 6025–6030

Field JK, Spandidos DA, Stell PM, Vaughan ED, Evan GI and Moore JP (1989) Elevated expression of the c-myc oncoprotein correlates with poor prognosis in head and neck squamous cell carcinoma. *Oncogene* **4**: 1463–1468

Goyal J, Smith KM, Cowan JM, Wazer DE, Lee SW and Band V (1998) The role for NES1 serine protease as a novel tumor suppressor. *Cancer Res* **58**: 4782–4786

Gramza AW, Lucas JM, Mountain RE, Schuller DE and Lang JC (1995) Efficient method for preparing normal and tumor tissue for RNA extraction. *Biotechniques.* **18**: 228

Ke LD, Adler-Storthz K, Clayman GL, Yung AW, Chen Z (1998) Differential expression of epidermal growth factor receptor in human head and neck cancers. *Head Neck* **20**: 320–327

Lang JC, Tobin EJ, Knobloch TJ, Bartynski KJ, Mountain RE, Nicholson R, De Young BR, Weghorst CM and Schuller DE (1998) Frequent mutation of p16 in squamous cell carcinoma of the head and neck. *Laryngoscope* **108**: 923–928

Liotta LA and Stetler-Stevenson WG (1991) Tumor invasion and metastasis**:** An imbalance of positive and negative regulation. *Cancer Res* **51**: 5054–5059

Loughran O, Clark LJ, Bond J, Baker A, Berry IJ, Edington KG, Ly IS, Simmons R, Haw R, Black DM, Newbold RF and Parkinson EK (1997) Evidence for the

 *© 2001 Cancer Research Campaign*

inactivation of multiple replicative lifespan genes in immortal human squamous cell carcinoma keratinocytes. *Oncogene* **14**: 1955–1964

Michalides R, Van Veelen N, Hart A, Loftus B, Wientjens E and Balm A (1995) Overexpression of cyclin D1 correlates with recurrence in a group of forty-seven operable squamous cell carcinomas of the head and neck. *Cancer Res* **55**: 975–978

Nawroz H, van der Riet P, Hruban RH, Koch W, Ruppert JM and Sidransky D (1994) Allelotype of head and neck squamous cell carcinoma. *Cancer Res* **54**: 1152–1155

Reed AL, Califano J, Cairns P, Westra WH, Jones RM, Koch W, Ahrendt S, Eby Y, Sewell D, Nawroz H, Bartek J and Sidransky D (1996) High frequency of p16 (CDKN2/MTS-1/INK4A) inactivation in head and neck squamous cell carcinoma. *Cancer Res* **56**: 3630–3633

Somers KD, Merrick MA, Lopez ME, Incognito LS, Schechter GL and Casey G (1992) Frequent p53 mutations in head and neck cancer. *Cancer Res* **52**: 5997–6000

Xia W, Lau YK, Zhang HZ, Liu AR, Li L, Kiyokawa N, Clayman GL, Katz RL and Hung MC (1997) Strong correlation between c-erbB-2 overexpression and overall survival of patients with oral squamous cell carcinoma. *Clin Can Res* **3**: 3–9

Yamaoka K, Masuda K, Ogawa H, Takagi K, Umemoto N and Yasuoka S (1998) Cloning and characterization of the cDNA for human airway trypsin-like protease *J Biol Chem* **273**: 11895–11901

*© 2001 Cancer Research Campaign*
